# Running after ghosts: are dead bacteria the dark matter of the human gut microbiota?

**DOI:** 10.1080/19490976.2021.1897208

**Published:** 2021-03-23

**Authors:** Sara Bellali, Jean-Christophe Lagier, Matthieu Million, Hussein Anani, Gabriel Haddad, Rania Francis, Edmond Kuete Yimagou, Saber Khelaifia, Anthony Levasseur, Didier Raoult, Jacques Bou Khalil

**Affiliations:** a IHU Méditerranée Infection, Marseille, France; b Aix-Marseille Université, IRD, MEPHI, Marseille, France, Marseille, France

**Keywords:** Human gut microbiota, cultured, not-yet-cultured, culturomics, metagenomics, fluorescence-activated cell sorting, viability, culturability

## Abstract

The human gut microbiota has been explored by a wide range of culture-dependent and culture-independent methods, revealing that many microbes remain uncharacterized and uncultured. In this work, we aimed to confirm the hypothesis that some of the species present in the human gut microbiota remain uncultured not because of culture limitations, but because all members of such species are dead before reaching the end of the gastro-intestinal tract.

We evaluate this phenomenon by studying the microbial viability and culturability of the human gut microbiota from the fresh fecal materials of eight healthy adults. For the first time, we applied fluorescence-activated cell sorting (FACS) combined with 16S metagenomics analysis and microbial culturomics.

We identified a total of 1,020 bacterial OTUs and 495 bacterial isolates through metagenomics and culturomics, respectively. Among the FACS metagenomics results, only 735 bacterial OTUs were alive, comprising on average 42% of known species and 87% of relative abundance per individual. The remaining uncultured bacteria were rare, dead, or injured.

Our strategy allowed us to shed light on the dark matter of the human gut microbiota and revealed that both metagenomics and culturomics approaches are needed for greater insight into the diversity and richness of bacteria in the human gut microbiota. Further work on culture is needed to enhance the repertoire of cultured gut bacteria by targeting low abundance bacteria and optimizing anaerobic sample conditioning and processing to preserve the viability of bacteria.

## Introduction

Almost one hundred and twenty years ago, it was postulated that there was a large discrepancy between initial cell counts and culturable bacteria on nutrient media from the same samples.^1^ This mismatch was subsequently been confirmed in several recent studies.^2–10^ The phenomenon was named “the great plate count anomaly” by Staley and Konopka in 1985.^8^ The fact that an overwhelming majority of micro-organisms, especially intestinal bacteria, do not grow on nutrient agar plates under laboratory conditions, led to the concept of “unculturable bacteria”, which is highly speculative, and “uncultured bacteria”. Furthermore, with the advent of culture-independent approaches and the dawn of metagenomics, it became clear that this “great plate count anomaly” resulted from a large number of unknown micro-organisms.^11,12^

Metagenomics has been widely used to describe the diversity of culturable and not-yet-cultured bacteria in the human gut microbiota.^13,14^ Indeed, only a fraction of intestinal bacteria can be accessed through standard cultivation techniques, although an estimated 80% are not-yet-cultured.^15^ Several hypotheses have been suggested to explain the microbial unculturability of microbes. For example, certain bacteria have low prevalence and/or low abundance, while others may require long incubation time to form visible colonies. Moreover, a great majority of intestinal bacteria are sensitive to oxygen, and some bacteria require specific nutrients and physical conditions for growth. In an attempt to solve this anomaly and increase our knowledge of the bacterial community composition in the human gut, recent advances in microbial culture techniques were applied to cultivate previously uncultured bacteria.^11,16–22^ One of the techniques that we have created to achieve this is “microbial culturomics”, a high-throughput culture method that has been developed in our lab. It consists of several culture conditions and nutrient media, followed by the identification of bacterial species using MALDI-TOF mass spectrometry and 16S rRNA gene sequencing.^23^ To date, culturomics has enabled the isolation of more than 300 new bacterial species in the human gut that were previously believed to be uncultured.^24^

Despite the advances in culture techniques, a huge number of gut bacterial species remain uncultured. However, culturomics provides viable pure cultures, unlike molecular approaches that only give information on the species level, without any information regarding their viability status. In addition, microbial culture using selective media has made it possible to detect and cultivate minority bacterial populations that may pass undetected by genomic technologies.^23^

In attempt to resolve the “uncultured” enigma of the intestinal bacterial species, and to describe the known richness and relative abundance of the live known and unknown bacterial population in human gut microbiota, we developed an optimized strategy using fluorescence-activated cell sorting (FACS) technique followed by16S metagenomics analysis. In addition, the metagenomics of FACS sorted live, injured, and dead bacterial populations, was compared for the first time to culturomics data, for the same samples. In this work, we aimed to test the hypothesis that some gut species remain uncultured not because of culture limitations, but because all members of such species are dead before reaching the end of the gastro-intestinal tract. In addition, among live species in feces, we compared cultured and not-yet-cultured species, and more specifically, we compared their abundance.

## Results

### Metagenomics analysis of sorted live, dead, and injured bacterial populations

#### Global analysis

**Figure 1.**
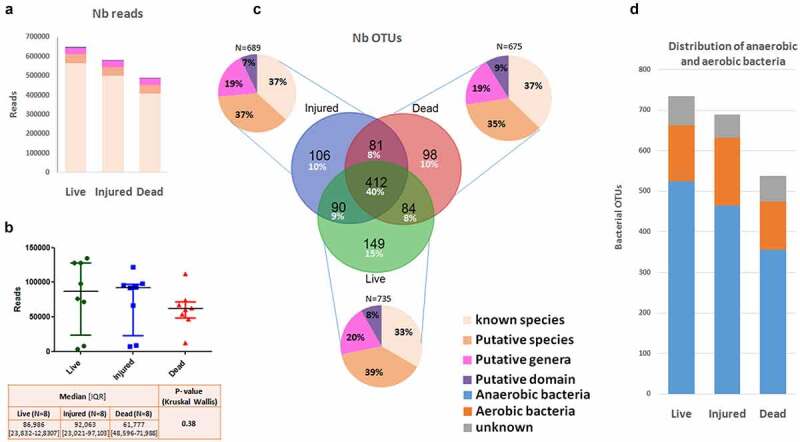
(a) Total read numbers in live, injured, and dead bacterial populations in eight fecal samples. (b) Graph representing Kruskal-Wallis test results on total number reads numbers in live, injured, and dead bacterial populations in eight fecal samples. (c) Pi chart representing the proportion of bacterial OTUs (known species, putative species, putative genera, and putative domain) in live, injured, and dead bacterial populations. (d) Distribution of anaerobic and aerobic bacteria in live, injured, and dead bacterial populations

Analysis of metagenomics sequencing from eight sorted fecal samples generated 2,287,729 reads in the live, injured, and dead bacterial populations that were organized into 3,590 unique OTUs (Operational Taxonomic Units) (Supplementary Table 1). Of these 3,590 bacterial OTUs, 2,569 (567,583 reads) were not assigned to any taxonomic level (unclassified). We subsequently excluded all unassigned OTUs and focused on bacterial OTUs assigned at least to the bacterial domain, which represented 1,020 unique bacterial OTUs (1,720,146 reads) (Supplementary Table 2). Ultimately, we obtained 648,460 reads (37.70%) generated from the viable bacterial population, 490,400 reads (28.51%) from the injured bacterial population, and 581,286 reads (33.79%) from the dead bacterial population (Figure 1a). There was no statistically significant difference in total reads between live, injured, and dead bacterial groups (Figure 1b). Regarding the total numbers of OTUs in the three bacterial populations, 15% (n = 149 OTUs) were exclusively live, 28% (n = 285 OTUs) were exclusively injured and/or dead, and 57% (n = 586 OTUs) were shared between live, injured, and dead bacterial populations (Figure 1c). In addition, anaerobic bacteria outnumbered aerobic bacteria by a factor of almost three in the live (n = 525 OTUs; 71.43%), dead (n = 356 OTUs; 66.17%) and injured (n = 465 OTUs 67.49%) bacterial population (Figure 1d).

Distinct differences at the phylum level were observed between live, injured, and dead bacterial groups. A total of 11 assigned phyla were identified, with four phyla dominating all samples in the live, injured, and dead bacterial groups. These were respectively, *Firmicutes* (35.11%, 46.12% and 46.01%), *Bacteroidetes* (33.65%, 13.33% and 16.96%), *Proteobacteria* (22.38%, 35.75% and 31.26%) and *Actinobacteria* (7.88%, 3.72 and 4.68%) (Supplementary Figure 1). No significant differences were observed between live, injured, and dead bacterial groups at the phylum level (Supplementary Figure 2).

**Figure 2.**
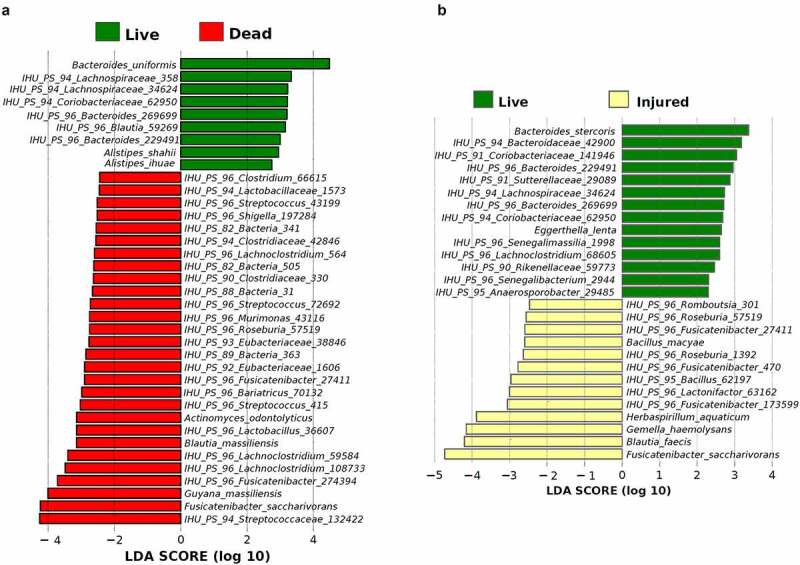
Histogram of the LDA scores computed for differentially abundant bacterial OTUs between live and dead bacterial groups (a) and live and injured bacterial groups (b)

We used linear discriminant analysis (LDA) of effect size (LEfSe) to compare the enrichment analysis of OTUs at all taxonomic levels between the live, injured, and dead bacterial populations (Figure 2). We found that the majority of dead bacterial OTUs were not previously cultured. Of these unknown bacteria, *IHU_PS_94_Streptococcaceae_132422* presented the highest LDA score (LDA score > 4.0) (Figure 2a). However, among known dead bacteria, *Guyana massiliensis* and *Fusicatenibacter saccharivorans* (LDA score ≥ 4.0) were overrepresented. The bacterium *F. saccharivorans*, was also enriched in the injured bacterial group. In addition, *Gemella haemolysans* and *Blautia faecis* had the highest LDA score (LDA score > 4.0) among the injured group (Figure 2b).

#### Live microbial richness and relative abundance of known species in fecal samples

**Figure 3.**
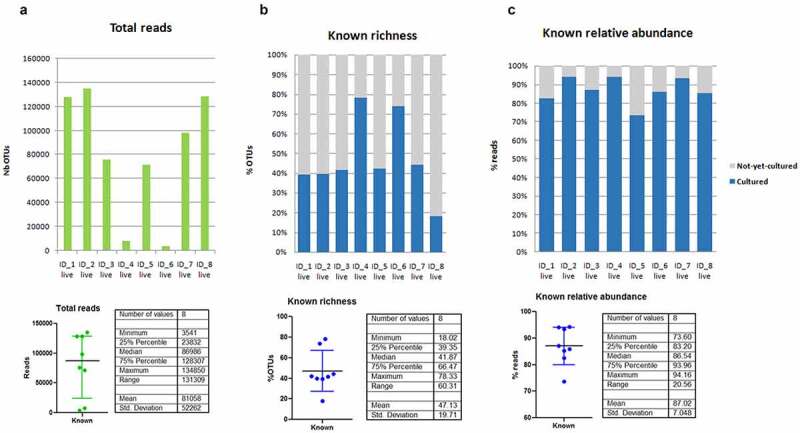
(a) **Total live read numbers** (known and unknown bacterial species) in eight fecal samples. (b) **Bacterial richness** of known and unknown bacterial species in the live bacterial population. (c) **Relative abundance** of known and unknown bacterial species in the live bacterial population

To quantify the richness and relative abundance corresponding to known species in each sample from the eight healthy donors, we excluded all dead and injured bacterial OTUs, and only considered OTUs from live bacteria (Figure 3a). We found 42% [83–94%] richness (Figure 3b) and 87% [39–66%] relative abundance corresponding to cultured species (Figure 3c) per individual (median [interquartile range]). These results suggest that majority species are more frequently cultured than minority species.

**Figure4.**
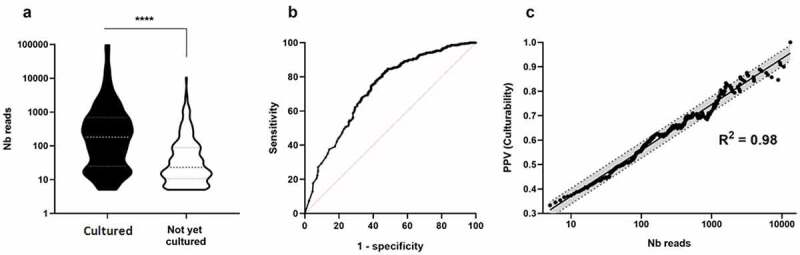
****: *p* < .00005. PPV: positive predictive value. (a)The number of reads was significantly higher for cultured than for not-yet-cultured species (bilateral Mann-Whitney test). Medians and interquartile ranges are shown. (b) **ROC curve** between culturability (cultured versus not-yet-cultured species) and abundance (total number of reads). (c) **Linear regression** of the culturability (positive predictive value to be “cultured”) versus the log10 of the total number of reads

Thus, we assess a putative relationship between culturability (probability of being cultured) and abundance (sum of reads for a species in the eight sampled individuals). The abundance of cultured species was significantly higher than the abundance of not-yet-cultured species (Figure 4a). Moreover, we found that species abundance followed a log-normal distribution for both cultured and not-yet-cultured species. Interestingly, the Receiver Operating Characteristic (ROC) analysis confirmed a highly significant relationship (*P* = 2.8 x 10^−23^) between the abundance of a species (number of reads) and its culturability (Figure 4b). The area under curves (AUCs) was 0.72 (95% confidence interval: 0.68–0.76). The ROC analysis also illustrated that species with total reads above 13,152 in the eight sampled individuals were all cultured (positive predictive value = 1 above 13,152 reads). Between 5 and 10,000 reads, the relationship between abundance and culturability was log-linear from 0.3 to 1 (linear regression after log10 transformation, R^2^ = 0.98, Figure 4c).

### Viability and culturability: metagenomics of sorted live, injured, and dead bacteria versus microbial culturomics

The microbial culturomics approach yielded 495 bacterial isolates (334 anaerobic, 161 aerobic) (Supplementary Table 3) from fresh fecal samples of eight healthy donors, using 58 culture media under anaerobic and aerobic conditions. In contrast, the metagenomics approach coupled with flow cytometry identified a total of 1,020 OTUs in the live, injured, and dead bacterial populations. Of them, 33.33% (340 OTUs; 1,470,844 reads) were assigned to known species, 37.35% (381 OTUs; 138,925 reads) were putative species, 20.29% (207 OTUs; 96,922 reads) were putative genus and 9.02% (92 OTUs; 13,455 reads) were putative domain.

**Figure 5.**
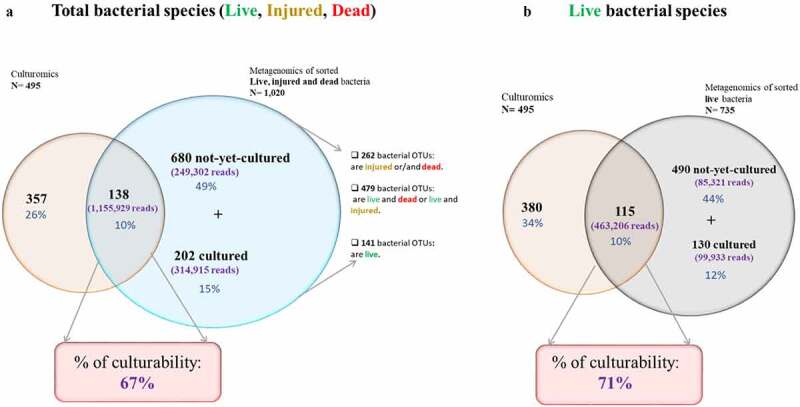
**Culturability and viability, percentages of culturable bacteria calculated from reads generated by metagenomics of sorted bacteria in eight fecal samples**: (a) Percentages of **culturable** bacteria calculated from total live, injured, and dead bacterial populations. (b) Percentages of **culturable** bacteria calculated from total live bacterial populations

We compared the bacterial populations cultivated by culturomics to metagenomics of sorted live, injured, and dead bacterial populations using a Venn diagram (Figure 5a). 357 cultured bacteria were exclusively identified by culturomics and 882 bacterial OTUs were exclusively identified by metagenomics. Of these missed bacteria (not isolated in the eight fecal samples using culturomics), 86 bacterial OTUs (34 anaerobic, 40 aerobic and 12 unknown bacterial OTUs) were exclusively dead, 141 bacterial OTUs (97 anaerobic, 22 aerobic and 22 unknown bacterial OTUs) were exclusively live, 99 bacterial OTUs (59 anaerobic, 31 aerobic and nine unknown bacterial OTUs) were exclusively injured and 556 bacterial OTUs were shared between live, dead and injured bacterial populations.

By comparing the metagenomics of live sorted bacteria and culturomics (Figure 5b), we found 115 known species (463,206 reads; 107 anaerobic, 8 aerobic) shared by both approaches. 380 known species (224 anaerobic, 158 aerobic) were exclusive to culturomics, and 620 bacterial OTUs (418 anaerobic, 130 aerobic and 72 unknown) were exclusively identified by metagenomics. Of these 620 bacterial OTUs (185,254 reads), 130 OTUs (99,933 reads) were assigned to known species (Figure 5). Of these 130 known species, 46 were isolated in previous culturomics studies, and 85 bacterial OTUs were not previously isolated using a culturomics approach, including 15 bacterial OTUs that were found in humans but not in the gut, 29 bacterial OTUs that were previously found to be associated with the human gut, and 41 bacterial OTUs that were not previously found in humans.

### Viability and culturability

Regarding the percentage of culturability, we determined the cultivable proportion of the human gut microbiota from total metagenomics reads of sorted live, injured, and dead bacteria. By including total bacterial OTUs (1,720,146 reads; 1,020 OTUs), the cultivable portion was 67% (1,155,929 reads) (Figure 5a). Moreover, when we only included live bacterial OTUs (648,460 reads; 735 OTUs), the percentage of culturability increased to 71% (463,206 reads) (Figure 5b).

Overall, comparing the culturomics and metagenomics of sorted live bacteria at the species level, culturomics (n = 495 bacterial isolates) doubled the number of isolated species compared to metagenomics (n = 245 known species). In addition, shared species were most abundant (463,206 reads; 115 known species) compared to the bacterial OTUs exclusively found by metagenomics (395,092 reads; 2322 OTUs).

## Discussion

The human gut microbiota harbors a wide range of micro-organisms which play different roles in human health and disease.^25^ Advances in next generation sequencing, in particularly advances in metagenomics, have provided great insights into the diversity of microbial populations in the human gut. Metagenomics claimed to be able to detect “uncultured” populations, nevertheless this technique is not able to determine whether the prokaryotes are alive or dead.^24^ Here, for the first time, we performed a fluorescence activated cell sorting (FACS) combined with 16S metagenomics analysis and microbial culturomics to explore the diversity and richness of the known and unknown bacterial species in the human gut microbiota of eight healthy individuals. In doing so, we successfully discriminated between live, injured, and dead bacterial groups using stringent methods that include negative and positive controls, unlike previous metagenomics analysis of the human gut.^13,14,23,26,27^ Our results showed that only about one third of reads were generated from the live bacterial population and the remaining bacteria were either injured or dead. Interestingly, within this live bacterial population, we found that known bacteria were the most abundant, so it will therefore be important to target minority populations using more specific culture media based on whole genome data along with a larger number of analyzed colonies.

This study revealed that the abundance of a species was a strong predictor of its culturability, with a biphasic relationship. This suggests that minority species constitute a large part of the dark matter of the live microbial population in human fecal samples. Future studies should include specific “kill the winner” strategies to isolate these minority species and/or use “high throughput” culturomics. These results also suggest that culturability is, at least in part, a stochastic or random process: the more colonies are identified, the more species would be found. In this way, single cell culturomics could be the next revolution using the same approaches developed for single cell sequencing: fluorescent-activated cell-sorting of individual cells collected by micromanipulation (serial dilution or nanotube), laser capture micro-dissection (LCM) and microfluidics.^28,29^

Moreover, by including total bacterial OTUs (live, injured and dead) at the genus level, we found that about 56% of bacterial OTUs were not-yet-cultured which is twice as high as previously estimated, 14 years ago.^15^ The same results were found by Sunagawa *et al*. using a single-cell genomics approach, where about 58% of gut species-level OTUs were not-yet-cultured.^27^ More recently, Nayefsh *et al*. found that 58% of gut species-level OTUs with sequenced genomes were uncultured species.^13^

One of the most important results of our study was that 28% of bacterial OTUs in the total fecal samples were exclusively found to be dead and/or injured. Of these non-live bacteria, about two-thirds were not-yet-cultured, and a large amount were anaerobic. This may explain why the majority of bacterial species were missed in culture, such as the genus *Romboutsia* (three bacterial OTUs). In agreement with our study, two recent studies compared the genomic profile of cultured and not-yet-cultured gut bacteria which revealed that uncultured species were missing genes encoding antioxidant and redox functions.^13,14^ These findings suggest that processing fecal samples under aerobic conditions may influence the mortality of these uncultured bacteria, and therefore their culturability. Recent studies have shown the impact of aerobic manipulations on the viability and diversity of fecal microbiota.^30–32^ Greater effort is needed to optimize anaerobic sample conditioning and processing, and consequently, anaerobic culturing.

We performed a comparative analysis between isolated cultures and FACS-sorted metagenomics for the same samples in order to explain the divergence between both approaches. The study led us to interesting conclusions. The first is that shared bacterial species between culturomics, and metagenomics represented only ten percent of the total bacteria and they are more abundant compared to the bacterial population identified by metagenomics alone. Secondly, the majority of bacterial species missing from culturomics were not-yet-cultured bacteria, and moreover, they were less abundant. Third, the percentage of culturability increased from 67% to 71% when we excluded dead and injured metagenomics data, which means that only a minority of bacterial species remain uncultured. And finally, at the species level, culturomics doubled the number of isolated species compared to metagenomics.

One limitation of our approach is the need to dilute the fecal sample to avoid background noise and achieve a successful bacterial sort. This may result in the loss of some rare bacterial populations. However, this could be enhanced by optimizing the settings of the flow cytometry cell sorter for efficient quantitative sorting, capable of analyzing more concentrated samples.

## Conclusions

We showed that fluorescence activated cell-sorting combined with 16S metagenomics analysis provides a way to distinguish between live, injured, and dead bacterial groups. Moreover, combining these culture-independent techniques to microbial culturomics, a valuable complementary approach, has enabled us to better understand the unculturability of some bacterial species. We aim to develop new culture techniques targeting rare bacteria that are still not-yet-cultured, as well as try filling the metagenomics gaps. Our process has made the link between culturomics and metagenomics. This bridge helped us answer many questions and resolve enigmas around the complementarity or discordance between the most widely used techniques to study the human gut microbiota.

## Materials & methods

### Stool sample collection

Fresh stool samples were obtained from eight healthy adults of different geographic origins: France (n = 2), Senegal (n = 2), Algeria (n = 2), Cameroon (n = 1) and Benin (n = 1) (Supplementary Table 4). All donors had lived in France for more than six months prior to sampling. They gave their written consent, and the project was approved by the ethics Committee of IHU Méditerranée Infection under number 2016–11. Donors had not received any antibiotics within the three months prior to sampling. Stool samples were collected in a sterile container and a GasPak generator (Becton Dickinson, Sparks, MD, USA) was immediately introduced. All containers were closed and kept in a plastic zipper bag until use.

### Microbial culturomics

**Figure 6.**
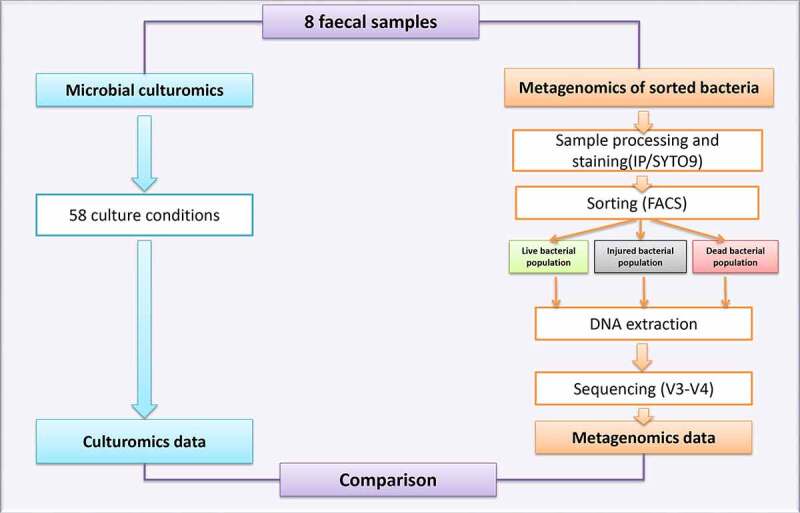
Workflow illustrating the overall strategy used in this study

The same eight fecal samples were cultured using 58 culture conditions that were detailed in a previous study carried out in our lab (Supplementary Table 5).^33^ We studied the bacterial culturability of these fecal samples by comparing the culturomics data to the data of the sorted metagenomics of live, injured, and dead bacterial populations for the same stool samples, as described below. The study strategy design is summarized in the flowchart represented in Figure 6.

### Metagenomics of sorted bacteria

#### Stool sample processing and staining conditions (BacLight staining)

Approximately 50 g of fecal sample were homogenized in 250 mL of normal saline solution (Fresenius Kabi, Sevres, France) using a commercial immersion blender (BOSCH Ultracompact 400 W) for five minutes. Fecal suspension was filtered using sterile coffee strainers to remove large aggregates. The slurry was centrifuged at 6,000 × g for 15 minutes, the supernatant was removed, and the pellet was re-suspended in 125 mL of normal saline solution. The homogenized stool solution was diluted with PBS (Life Technologies, Paisley, United Kingdom) to 1/1000. The staining step was carried out using the Live/Dead BacLight kit (Invitrogen, Eugene, USA) as per the manufacturer’s recommendation: 1 mL of diluted fecal sample at the 10^−3^ dilution, was stained with 1 µL of SYTO9 and 1 µL of propidium iodide (PI) in a microbial safety cabinet and then incubated for 15 minutes in the dark at room temperature, before being processed in a cell FACS sorter (BD Biosciences) and then sequenced.

#### Flow cytometry and cell sorting

Live and dead dye optimization and protocols for live and dead assay were previously described by Bellali *et al*.^32^ We proceeded to analyze and sort artificial mixed fractions, in order to assess the fecal samples. We used a BD FACS Aria™ Fusion Special Order (SORP) cell sorter cytometer (BD Biosciences) equipped with a combination of six lasers (355 nm, 405 nm, 488 nm, 561 nm, and 640 nm) in a biosafety cabinet for the best safety performance and sorting results. The flow sheath was 0.22 μm in-line filtered. The pressure was stabilized for at least one hour before experiments began. A photodiode was used as a standard FSC-detector on this instrument, and triggering was based on the side scatter. Sorting was performed using a 70 μm nozzle, pressure of 70 PSI and a frequency of 90 kHz, after discriminating between all populations based on forward scatter, side scatter, SYTO9 and PI. To analyze the purity of the sorted fraction, collected samples were processed under the same conditions. The purity calculation was based on the ratio between the different population counts, by applying the following equation: [% Sorted fraction = (#Sorted fraction/# all fractions) x 100] after reanalysis of the sorted population. We managed to differentiate between three populations clearly separated on the dot plots, “live” bacterial cells (SYTO9- stained), “dead” bacterial cells (IP-stained), and “injured” or damaged bacterial cells (IP and SYTO9-stained) between the live and the dead bacterial populations. All three populations were sorted and then sequenced as described below. The efficiency and purity of our sorting was validated by culturing the sorted fractions in Columbia sheep blood agar plates (BioMérieux, Marcy l’Etoile, France) under aerobic and anaerobic conditions for 48 hours at 37°C, in order to demonstrate that dead and live bacteria had been correctly discriminated, and that there was no cross-contamination.

#### DNA extraction and metagenomics sequencing

DNA was extracted from all the dead, live and injured bacterial populations. Briefly, sorted samples were completely centrifuged and re-suspended in 750 µL of 0.22 micro-filtered PBS. DNA was then extracted according to the extraction methods 1 and 5 described by Angelakis *et al*.^34^

The extracted fractions (live, dead and injured) were then sequenced for 16S rRNA on MiSeq technology (Illumina, Inc, San Diego CA 92121, USA) as previously described by Angelakis *et al*.^34^

#### Metagenomics analysis

The paired-end reads were assembled into contigs using Pandaseq.^35^ The high-quality sequences containing both primers (forward and reverse) were then selected for the next step. At the filtering stage, all sequences containing N and sequences which were shorter than 200nt were removed. Sequences longer than 500nt were trimmed. In addition, the forward and reverse primers were also removed from each of the sequences. To remove the chimeric sequences we applied an additional filtering step using UCHIME^36^ by USEARCH.^37^ The filtering steps were carried out using the QIIME pipeline.^38^ The clustering of duplicate sequences (the dereplication process) was carried out on the filtered sequences, and they were subsequently sorted by decreasing abundance.^39–41^ The clustering of OTUs was performed with 97% identity for each metagenome. The Silva SSU and LSU database and release 132 from the Silva website were downloaded and integrated. From this, a local database of predicted amplicon sequences was built by extracting the sequences containing both primers.16S sequences from 375 species isolated in our laboratory from diagnosis or culturomics were added to the database (Supplementary Table 6). A reference database of 14,459 sequences was thus generated. All the putative species of previous analysis were also added. Finally, the generated database contained 76,368 sequences ready to be used. Regarding taxonomic assignments, we applied at least five reads per OTU because our samples were diluted at 1/1,000, and after sorting we had 1,000,000 cells per tube for each population: live, dead, and injured bacteria. The OTUs were then searched against each database using BLASTN.^42^ The sequences were assigned a taxonomic classification using the criteria of ≥ 77% identity for domain, ≥ 75% identity for phylum, ≥ 80% identity for class, ≥ 85% identify for order, ≥ 90% identity for family, ≥ 94% identity for genus and ≥ 97% sequence identity for species. The best match of ≥ 97% identity and 100% coverage for each of the OTUs was extracted from the reference database, and taxonomy was assigned up to the species level. Sequences identities below 77% were assigned to the “unclassified or unassigned” category. Finally, we counted the number of OTUs.

## Statistical analysis

Differentially abundant OTUs between live, injured, and dead sorted bacteria were identified using the LDA Effect Size (LEfSe: Linear Discriminant Analysis Effect Size) algorithm available online at (http://huttenhower.sph.harvard.edu/galaxy/root)^43^. The threshold on the logarithmic discriminant analysis (LDA) score was set to 2.0 and the significance level was 0.05.

To test whether there was a relationship between “cultured species” and “not-yet-cultured species” and their abundance in the human gut microbiota, we used a ROC analysis and the area under curve was calculated. Any species previously reported cultured in the literature or in our culturomics approach was defined as “cultured”. OTUs in the “live” population that do not correspond to any cultured species, were defined as “not-yet-cultured species”. The quantitative variable for microbial abundance was the total reads (sum of reads in the eight fecal samples for each species). A comparison of read numbers between live, injured, and dead bacterial group was carried out using the non-parametric Kruskal-Wallis test. Statistical analyses were performed using XLSTAT 2019.3.1 (Addinsoft, Paris, France) and Graph Pad (Prism v8.2.1) (GraphPad Software, San Diego, California USA).

## Supplementary Material

Supplemental Material

## Data Availability

All data generated or analysed during this study are included in this published article and its supplementary information files.
