# Supplementary material for: Running after ghosts: are dead bacteria the dark matter of the human gut microbiota?
Source: Gut Microbes. 2021 Mar 23;13(1):1897208. doi: 10.1080/19490976.2021.1897208 (PMC7993147; doi:10.1080/19490976.2021.1897208)
Supplement: Supplemental Material [file KGMI_A_1897208_SM1810.zip › Supplementary information/Supplementary caption.docx]

**Additional files**

**Supplementary Figure 1:** Relative read abundance of the bacterial phyla found in the live, injured, and dead bacterial populations in the eight faecal samples.

**Supplementary Figure 2:** Top four predominant phyla in live, injured, and dead bacterial populations (*Actinobacteria, Bacteroidetes, Firmicutes and Proteobacteria).* Graphics and table represent Kruskal-Wallis test results on the total number of reads in eight faecal samples.

**Supplementary Table1:** Total bacterial reads including unclassified bacterial OTUs generated from FACS sorted live, injured, and dead bacterial populations from eight healthy donors. All bacterial species were classified according to their domain, phylum, order, class, family, genus, and species, and according to their tolerance to oxygen (0 being able to tolerate oxygen and 1 being strictly anaerobic).

**Supplementary Table2**: Total bacterial OTUs and reads excluding unclassified bacterial OTUs generated from FACS sorted live, injured, and dead bacterial populations from eight healthy donors. All bacterial species were classified according to their domain, phylum, order, class, family, genus, and species, and according to their tolerance to oxygen (0 being able to tolerate oxygen and 1 being strictly anaerobic).

**Supplementary Table 3**: Taxonomic classification (domain, phylum, order, class, family, genus, and species) of total isolates identified by culturomics approach classified according to their tolerance to oxygen tolerance (0 being able to tolerate oxygen and 1 being strictly anaerobic).

**Supplementary Table 4**: Characteristics of donors who provided faecal samples used in this study.

**Supplementary Table 5**: List of culture conditions and media used.

**Supplementary Table 6**: List of the 375 species isolated in our laboratory from diagnosis and culturomics added in the IHU culturomics database.

**Supplementary Table 7**: Total reads and bacterial OTUs including cultured, uncultured, and unassigned bacterial OTUs generated from FACS sorted live, injured, and dead bacterial populations.

**Supplementary Table 8**: Total bacterial OTUs exclusively assigned to the species level generated from FACS sorted live, injured, and dead bacterial populations. All bacterial species were classified according to their domain, phylum, order, class, family, genus, and species.

**Supplementary Methods and Results**: Additional analysis using DADA2 software.
