# Supplementary figures and images for: Running after ghosts: are dead bacteria the dark matter of the human gut microbiota?
Source: Gut Microbes. 2021 Mar 23;13(1):1897208. doi: 10.1080/19490976.2021.1897208 (PMC7993147; doi:10.1080/19490976.2021.1897208)

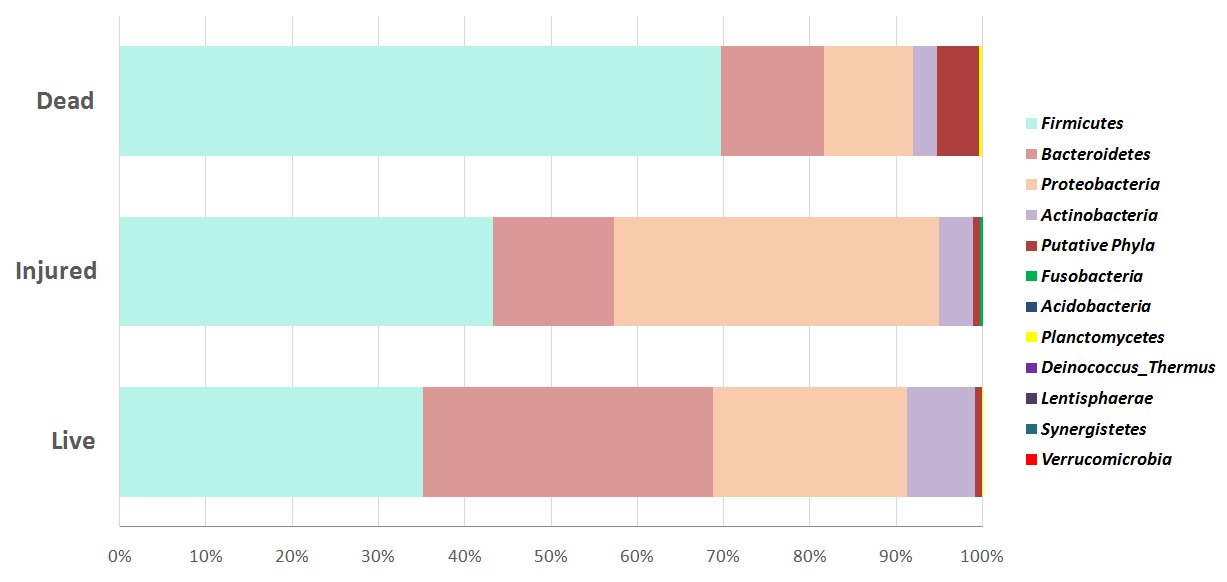

Supplement: Supplemental Material [file KGMI_A_1897208_SM1810.zip › Supplementary information/Supplementary Figure 1.tif]

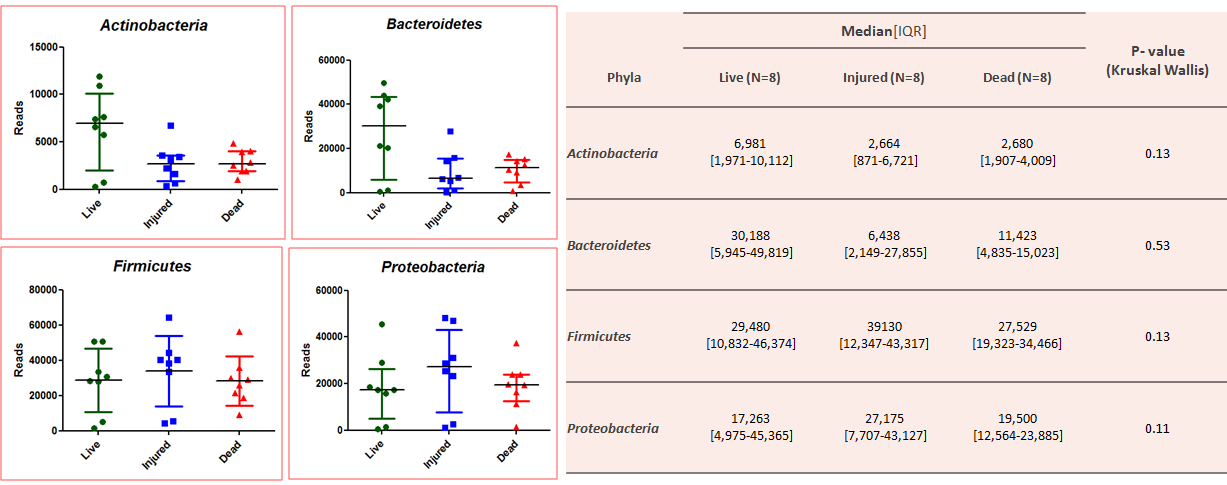

Supplement: Supplemental Material [file KGMI_A_1897208_SM1810.zip › Supplementary information/Supplementary Figure 2.tif]
